# Avoiding treatment bias of REDD+ monitoring by sampling with partial replacement

**DOI:** 10.1186/s13021-015-0020-y

**Published:** 2015-05-08

**Authors:** Michael Köhl, Charles T Scott, Andrew J Lister, Inez Demon, Daniel Plugge

**Affiliations:** 1 University of Hamburg, Institute for World Forestry, Leuschnerstrasse 91, 21031 Hamburg, Germany; 2 USDA Forest Service, Northern Research Station, Forest Inventory and Analysis, 11 Campus Blvd, Suite 200, Newtown Square, PA 19073 USA; 3 Centre for Agricultural Research in Suriname (CELOS), Postbus 1914, Paramaribo South, Suriname

**Keywords:** Measurement, Reporting and verification (MRV), Forest carbon stock and carbon stock change estimation, Representativeness over time

## Abstract

**Background:**

Implementing REDD+ renders the development of a measurement, reporting and verification (MRV) system necessary to monitor carbon stock changes. MRV systems generally apply a combination of remote sensing techniques and in-situ field assessments. In-situ assessments can be based on 1) permanent plots, which are assessed on all successive occasions, 2) temporary plots, which are assessed only once, and 3) a combination of both. The current study focuses on in-situ assessments and addresses the effect of treatment bias, which is introduced by managing permanent sampling plots differently than the surrounding forests. Temporary plots are not subject to treatment bias, but are associated with large sampling errors and low cost-efficiency. Sampling with partial replacement (SPR) utilizes both permanent and temporary plots.

**Results:**

We apply a scenario analysis with different intensities of deforestation and forest degradation to show that SPR combines cost-efficiency with the handling of treatment bias. Without treatment bias permanent plots generally provide lower sampling errors for change estimates than SPR and temporary plots, but do not provide reliable estimates, if treatment bias occurs, SPR allows for change estimates that are comparable to those provided by permanent plots, offers the flexibility to adjust sample sizes in the course of time, and allows to compare data on permanent versus temporary plots for detecting treatment bias. Equivalence of biomass or carbon stock estimates between permanent and temporary plots serves as an indication for the absence of treatment bias while differences suggest that there is evidence for treatment bias.

**Conclusions:**

SPR is a flexible tool for estimating emission factors from successive measurements. It does not entirely depend on sample plots that are installed at the first occasion but allows for the adjustment of sample sizes and placement of new plots at any occasion. This ensures that in-situ samples provide representative estimates over time. SPR offers the possibility to increase sampling intensity in areas with high degradation intensities or to establish new plots in areas where permanent plots are lost due to deforestation. SPR is also an ideal approach to mitigate concerns about treatment bias.

## Background

In November 2013, the nineteenth session of the Conference of the Parties (COP 19) agreed on the “Warsaw Framework for REDD Plus”, which consists of seven decisions relating to the implementation of REDD+ [1]. Together with the UNFCCC Cancun Agreements [2] and Durban outcomes [3], these decisions are a major step forward for the implementation of REDD+ at the national level. According to decision 11/CP.19 [1], the modalities for national forest monitoring systems should in the full implementation phase provide data and information that are transparent, consistent over time, suitable for measurement, reporting and verification (MRV) and build upon existing systems while being flexible and allowing for improvement [1]. In accordance with national circumstances and respective capabilities, robust and transparent national forest monitoring systems are to be developed (Decision 4/CP.15) [4].

MRV systems as an integral part of REDD+ implementation mainly focus on the assessment of carbon stock change. Decisions to adopt specific operational systems at national and local levels are subject to the country’s unique circumstances, such as differences in forest types, drivers of deforestation and forest degradation, or livelihood impacts. Therefore, finding an operational approach that adheres to the international (IPCC) requirements is a matter of much debate.

MRV systems generally apply a combination of remote sensing techniques and in-situ field assessments to provide information on activity data and emission factors. The current study focuses on in-situ assessments and addresses the effect of treatment bias, which is introduced by managing forests on permanent sampling plots differently than the surrounding forests.

**Figure 1:**
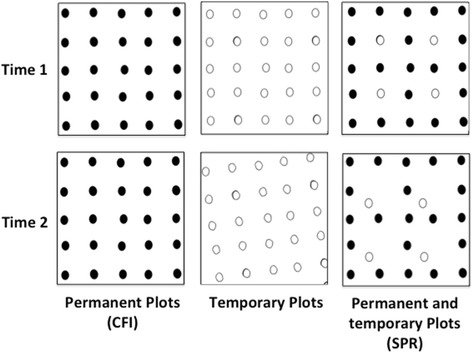
Sampling at successive occasions (filled circles are permanent plots and hollow circles are temporary plots).

In the scope of forest resource assessments, several approaches have been developed for the estimation of forest change. These generally fall into three categories: 1) the same sampling units are assessed at each occasion (permanent plots), 2) new sampling units are selected at each occasion (temporary plots), or 3) a mixture of permanent and temporary plots is applied (Figure 1).

A major obstacle to the use of permanent plots is the likelihood that they will become non-representative due to treatment bias [5]. Land management on permanent plots with known locations might differ from that of the surrounding forests. When payments are linked to the results of field assessments, the potential for treatment bias can be high. For example, activities like tree cutting that would normally be classified as forest degradation could be deliberately excluded from areas on or around permanent plots in order to maintain biomass and secure payments. For an operational and sound MRV system, it is critical that the shortcomings of inventory designs are not exploited for the generation of (unjustified) financial benefits. Therefore, MRV systems have to be immune to treatment bias and thus produce objective stock change estimates.

It has been shown that permanent sample plots guarantee the highest precision for change estimates [6-8]. Continuous Forest Inventory (CFI), or the use of permanent plots in forestry, was introduced in the middle of the last century [9-11]. In this design, the number of sampling units and their allocation is determined at inventory establishment and retained over time. Therefore, CFI designs show a limited ability to adapt to changing population conditions. This holds especially true in the scope of REDD+. Due to deforestation, forested permanent sample plots might become deforested over time and thus sampling intensity might become too low to provide forest change estimates with a desired reliability. Although an increase of sampling intensity is called for in locations with high degradation activities, the use of permanent plots selected with fixed selection probabilities precludes this type of adaptation. This inflexibility, together with the potential for treatment bias, is a considerable disadvantage compared to alternatives.

Temporary plots are an alternative to permanent plots. At each occasion, plots are allocated independently from assessments at previous occasions, allowing for a flexible adjustment of sampling intensities over time. As an individual plot is assessed only once, treatment bias is not an issue. However, one major disadvantage of temporary plots is the high sampling error associated with the estimation of change. Avoidance of observer bias by utilizing temporary plots only is therefore achieved at the expense of a loss in precision and reliability, making designs based on temporary plots less cost-efficient than those with an equal number of permanent plots [12].

The desire to exploit the advantages and avoid the disadvantages of temporary and permanent plots motivated the development of a sampling approach that combines both approaches. In this design, a subset of the plots allocated at inventory establishment are remeasured (permanent plots), and the remaining subset is replaced by new, temporary plots. This proceeds in repeated inventories in an alternating fashion over successive inventory cycles. The procedure is known as Sampling with Partial Replacement (SPR) and was introduced into forestry by Ware and Cunia [13]. Scott [14] presented a sample-based estimator that combines the variance from the permanent (matched) and temporary (unmatched) plots for change estimation. SPR has been recommended as a flexible tool to meet precision requirements of current forest status and trend estimates in a cost-efficient way [13-15].

This paper will present the statistical background of sampling on successive occasions in the scope of MRV. A descriptive example is used to illustrate the procedure and identify the pros and cons of the 3 alternative sampling approaches. It is demonstrated how SPR can be used in MRV systems in order to mitigate problems such as treatment bias and loss of optimality of sample intensity over time with designs using permanent plots, and provide a cost-effective improvement over designs using only temporary plots.

## Results and discussion

The performance of the 3 forest change assessment design alternatives

- Continuous forest inventory (CFI) design, utilizing permanent (remeasured) plots only
- Temporary plot design, where an independent sample is drawn at each occasion
- Sampling with Partial Replacement (SPR) design, utilizing a mixture of permanent and temporary plots

is illustrated by a set of permanent plots located in the Suriname’s forest belt for change estimation under different deforestation and degradation scenarios. The permanent plots were established in the late 1970’s, when several silvicultural treatments with different logging intensities were applied. Since then, no forest management has taken place. For our study we utilized the observations from the most recent measurements, which were made in 2000 and in 2013. On 750 plots with a size of 400 m^2^ different degradation and deforestation intensities were simulated by selectively removing certain trees based on diameter thresholds.

To assess the effects of the alternative sampling designs, 6 scenarios that reflect realistic deforestation and degradation activities (including no intervention) were created. For a subset of the plots (5% to 20%), different levels of treatment bias were simulated on the plot level. Treatment bias was simulated by deliberately excluding the permanent plots from being affected by forest loss or degradation that occurred elsewhere in the study area, leading to a non-representative sample. For scenarios with no treatment bias applied, simulated deforestation and degradation occurred on permanent plots with the same frequency at which it occurred elsewhere, leading to a representative sample.

**Figure 2:**
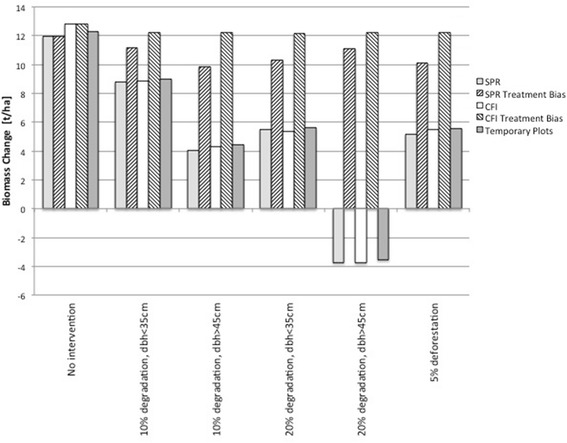
Biomass change under different scenarios.

**Figure 3:**
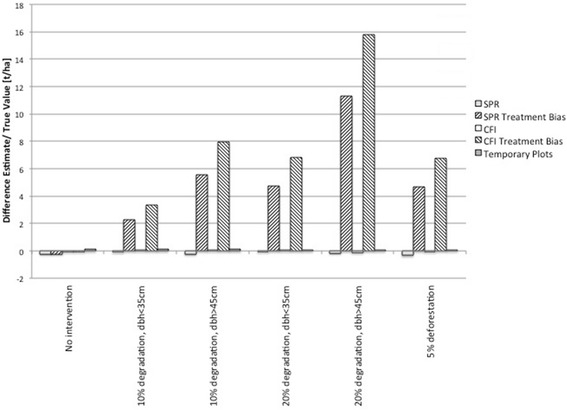
Difference between estimated and true biomass changes.

**Figure 4:**
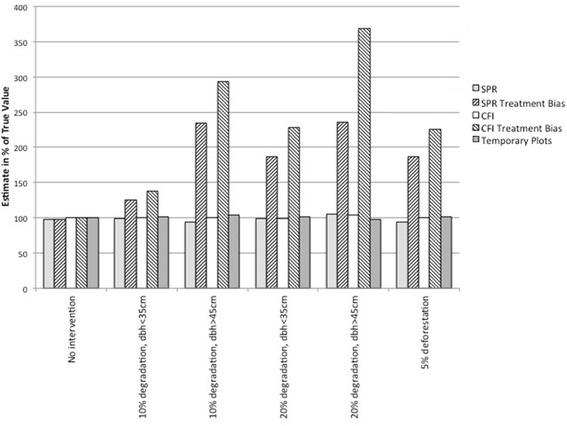
Estimate in percent of true change.

**Figure 5:**
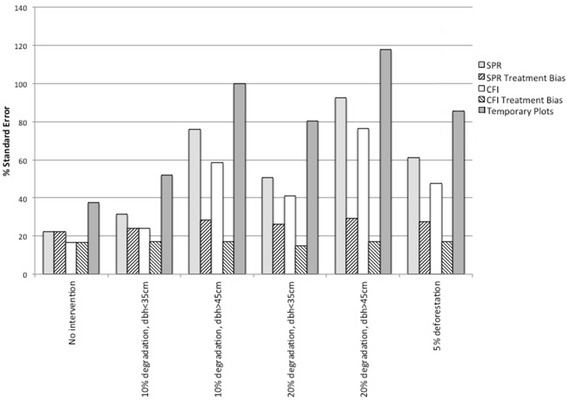
Percent standard error of change estimates.

The following results are based on a Monte Carlo simulation with 1000 iterations for each sampling design and scenario combination. For each combination, biomass change (Figure 2), the absolute (Figure 3) and percent (Figure 4) difference between estimated biomass changes and true population values, and the percent standard error (Figure 5) were calculated. We emphasize that the results are strongly influenced by the characteristics of the underlying forest population. They reveal the general behavior of the design alternatives under different degradation and deforestation patterns, but cannot be generalized for any forest population as biomass changes depend of the forest composition and structure as well as on the specific degradation and deforestation regimes. However, the case study presented provides insight in the understanding of the general behavior of the 3 sampling design approaches.

The absolute value of biomass loss differs by scenario (Figure 2). Degradation and deforestation lead to a decreasing total biomass, but only under heavy degradation activities where on 20% of the forest area all trees with dbh >45 cm were removed the growth of the remaining forest could not compensate for the biomass loss by degradation activities. Treatment bias consistently leads to an overestimation of biomass changes and does not capture the true development. Under a REDD+ regime this would result in unjustified benefits. A rogue stakeholder could use this effect to manipulate carbon budgets.

The absolute (Figure 3) and percent (Figure 4) difference between estimated biomass changes and true population values illustrate the effects of treatment bias. Treatment bias results in substantial overestimation of biomass gains; the overestimation is higher for CFI than for SPR, as SPR utilizes some new (temporary) plots at the second occasion, which replace some of those plots from treatment bias. Where only temporary plots are utilized assessments are not subject to treatment bias as the location of the location of the set of plots installed at the second occasion is not known in advance.

Figure 5 presents the percent standard errors for the estimation of biomass change under the different scenarios and sampling design alternatives. Beside population variability the standard errors in change estimates are generally affected by two variance components: (1) the variance introduced by biomass growth, which is influenced by stand structure and site quality, and (2) the variance due to disturbances, i.e., degradation and deforestation. The second variance component is not present when plots are subject to treatment bias. Thus the percent standard errors of CFI and SPR under treatment bias show comparable magnitudes, as they refer to similar, non-disturbed biomass development.

Where no treatment bias is present, the variability introduced by disturbances inflates the resulting standard errors. CFI results in the smallest standard errors, as the estimator utilizes the covariance between observations at successive occasions (Eq. 3). Temporary plots show the largest sampling errors; the standard errors obtained by SPR, which apply an update of unremeasured plots based on the regression relationship with remeasured plots (Eq. 4), are at an intermediate level.

Treatment bias considerably affects change estimates and their standard errors. Even substantial biomass losses outside the remeasured plots remain undetected. This is reinforced by the fact that standard errors of treatment-biased change estimates ignore the presence of degradation and deforestation activities and thus remain small due to the pronounced correlation between plot values on successive occasions.

## Conclusions

MRV systems for REDD+ aim at the provision of consistent and reliable estimates of biomass and carbon stock changes at successive occasions. As the nature of these estimates affects a country’s financial benefits associated with participation in REDD, it is important that inventory methods are subject to careful validation.

It is a widespread practice in forestry to utilize permanent plots for change estimates. However, permanent plots are subject to treatment bias as they may be excluded from degradation or deforestation activities once their location is known. This opens the potential for non-representative samples and associated estimates due to either honest mistakes or fraudulent activities. Biased estimates linked with small sampling errors have an unknown level of risk where only permanent plots are used. Since one of the main goals of an MRV is to accurately characterize the carbon dynamics of an area of interest, using solely permanent plots can thus call into question the scientific validity of an MRV system if treatment bias is not controlled.

Given the absence of a selection bias the problem of non-representativeness is not a concern when temporary plots are used. However, an assessment built on only temporary plots will result in substantial sampling errors and low cost-efficiency. SPR, on the other hand, offers a solution to both low cost-efficiency and potential treatment bias, as it combines temporary and permanent plots. SPR can be used to guard against treatment bias on permanent plots and improves the reliability of change estimates.

Another concern about MRV systems based solely on permanent plots is the determination of sample size and plot location at inventory establishment. This design lacks flexibility because the set of plots installed at the first occasion has to be remeasured at successive occasions, regardless of changes that occur on the landscape. This holds especially true in situations where forest plots are lost due to deforestation activities or where degradation activities are shifting.

Under REDD+ monitoring the application of SPR offers compelling advantages over designs based solely on permanent plots. SPR designs are flexible as new plots can be established at any occasion and old plots can be replaced by new plots whenever necessary. The SPR estimation procedures help detect and guard against treatment bias and can generate cost-effective estimates of forest carbon dynamics as well as help provide verification for the scientific validity of change estimates.

Where degradation is concentrated in specific regions, SPR can be combined with stratification for further reductions in sampling error [15]. Stratification rules can be designed that incorporate the magnitude of degradation intensities and utilize auxiliary information e.g. from remote sensing [16,17]. In each stratum an independent SPR design can be applied and the number of remeasured and temporary plots can be optimized [18,19].

## Methods

### State of the art

In REDD+ monitoring, estimates of both current values and change of biomass and associated carbon stock are of interest. Change estimates are generally obtained by

1 $$ \widehat{C}=\widehat{Y}-\widehat{X} $$

where

$\widehat{X}$ = first occasion (time 1) estimate of the mean, $\widehat{X}=\frac{{\displaystyle {\sum}_{i=1}^{n_1}}{X}_i}{n_1}$

*Ŷ* = second occasion (time 2) estimate of the mean, $\widehat{Y}=\frac{{\displaystyle {\sum}_{i=1}^{n_2}}{Y}_i}{n_2}$

*X*_*i*_ = measurement on plot *i* at time 1, *i* = 1,… *n*_*1*_

*Y*_*i*_ = measurement on plot *i* at time 2, *i* = 1,… *n*_*2*_

*n*_*1*_ = number of plots at time 1

*n*_2_ = number of plots at time 2

If samples at the 2 occasions are selected independently, then these temporary plots do not match between the 2 occasions. With the application of temporary plots the variance of $\overline{C},\kern0.37em V\left(\overline{C}\right)$ is obtained by

2 $$ {V}_t\left(\widehat{\overline{C}}\right)=V\left(\widehat{\overline{Y}}\right)+V\left(\widehat{\overline{X}}\right) $$

where

$$ V\left(\widehat{\overline{X}}\right)= variance\  of\ \widehat{\overline{X}}=\frac{{\displaystyle {\sum}_{i=1}^{n_1}}{\left({X}_i-\widehat{\overline{X}}\right)}^2}{n_1\left({n}_1-1\right)} $$

$$ V\left(\widehat{\overline{Y}}\right)= variance\  of\ \widehat{\overline{Y}}=\frac{{\displaystyle {\sum}_{i=1}^{n_2}}{\left({Y}_i-\widehat{\overline{Y}}\right)}^2}{n_2\left({n}_2-1\right)} $$

If only the current status of the resource is to be considered, temporary sample plots are often more cost effective than permanent plots, since there are no required expenditures for monumenting the sample plot centers and the registration of sample tree locations. If change has to be estimated, however, sampling errors are lower using permanent sample plots, since the difference between 2 independent observations is not caused by change alone, but also by the variation within the 2 populations. The estimated variance of change from a permanent plot design is given by

3 $$ {V}_{CFI}\left(\widehat{\overline{C}}\right)=V\left(\widehat{\overline{Y}}\right)+V\left(\widehat{\overline{X}}\right)-2r\sqrt{V\left(\widehat{\overline{Y}}\right)}\sqrt{V\left(\overline{\widehat{X}}\right)} $$

where *r* is an estimate of the correlation coefficient between the observations of the second and the first occasion. The higher the correlation is between paired observations from the first and second inventory, the smaller is the variance of their difference. Therefore, for the same cost, permanent plots lead to a smaller sampling error than temporary plots for the estimated change.

The CFI method, despite its obvious advantage, encounters practical and inferential problems. Over time the locations of sample plots may become known beyond the surveyors and, as a result, they may be deliberately treated differently than the surrounding forest. This non-trivial risk is especially acute for visibly marked sample plots. The latent potential of an inferential problem therefore exists because, as paraphrased by [20], “there is no guarantee that sample plots, visible or not, will remain representative of the target population”. Inventories that potentially don’t represent reality will lose credibility. This holds especially true for inventories in the scope of REDD+; non-representative treatment applied on permanent plots could corrupt the reliability of emission estimates.

Those problems can be controlled and mitigated by Sampling with Partial Replacement (SPR), which utilizes a mixture of both permanent and temporary plots. New, temporary plots established at the second and every following occasion can be utilized to assess the potential for treatment bias on the remeasured subset. In addition, plots that are lost due to land-use change can be “replaced” by new plots by increasing the sampling intensity on the new set of permanent plots so that the number of forested plots does not diminish over time.

SPR was introduced into forest inventory around 1960 [13,21]. Scott [14] presented a consistent set of estimators for SPR, which will be presented in the following.

**Figure 6:**
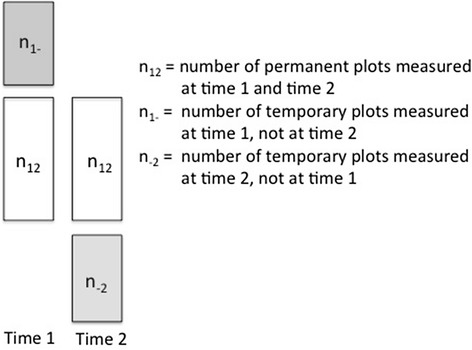
Types of sample plots used for Sampling with Partial Replacement for 2 occasions.

For 2 occasions, 3 types of sample plots can be considered (Figure 6):

- Sample plots that are measured on the first occasion as well as on the second occasion (permanent, matched sample plots referred to as the *n*_*12*_ sample).
- Sample plots that are only measured on the first occasion (unmatched plots referred to as the *n*_*1−*_ sample).
- Sample plots that are only measured on the second occasion (new, unmatched plots referred to as the *n*_*−2*_ sample).

The SPR estimation procedures involve 4 steps [14]:

1. The current state is obtained by 2 means. One mean is based on the measurements of permanent (remeasured) plots and the updated values of the temporary (not remeasured) plots (Eq.4). The first mean, ${\widehat{\overline{Y}}}_{12}$, is formed by updating the time 1 mean using the simple linear regression between time 1 and 2 on the remeasured plots. This regression, in effect, updates the values of the sample plots that are not remeasured (*Y*_*1*−_). A second mean is derived from the new (temporary) sample plots (Eq.5).

4 $$ {\widehat{\overline{Y}}}_I={\widehat{\overline{Y}}}_{12}+{\widehat{\beta}}_{YX}\left({\widehat{\overline{X}}}_1-{\widehat{\overline{X}}}_{12}\right) $$

5 $$ {\widehat{\overline{Y}}}_{II}={\widehat{\overline{Y}}}_{-2}=\frac{{\displaystyle {\sum}_j^{n_{-2}}}{Y}_{-2j}}{n_{-2}} $$

where

$$ {\widehat{\overline{X}}}_1=\mathrm{time}\ 1\ \mathrm{mean}\ \mathrm{of}\ \mathrm{all}\ \mathrm{plots}\ \mathrm{assessed}\ \mathrm{at}\ \mathrm{time}\ 1 $$

$$ {\widehat{\overline{X}}}_{12}=\mathrm{time}\ 1\ \mathrm{mean}\ \mathrm{of}\ \mathrm{all}\ \mathrm{permanent}\ \left(\mathrm{remeasured}\right)\ \mathrm{plots} $$

$$ {\widehat{\overline{Y}}}_{12}=\mathrm{time}\ 2\ \mathrm{mean}\ \mathrm{of}\ \mathrm{all}\ \mathrm{permanent}\ \left(\mathrm{remeasured}\right)\ \mathrm{plots} $$

$$ {\widehat{\overline{Y}}}_{-2}=\mathrm{mean}\ \mathrm{of}\ \mathrm{all}\ \mathrm{time}\ 2\ \mathrm{temporary}\ \mathrm{plots} $$

$$ {Y}_{-2\mathrm{j}}=\mathrm{measurement}\ \mathrm{of}\ \mathrm{time}\ 2\ \mathrm{temporary}\ \mathrm{plots}\ j,\ j=1,\dots,\ {n}_{-2} $$

${\widehat{\beta}}_{YX}$ = slope coefficient in the simple linear regression of *Y*_*12*_ on *X*_*12*_ = $\frac{s_{XY}}{s_{X12}^2}$

$$ {s}_{XY}=\frac{{\displaystyle {\sum}_j^{n_{12}}}\left({Y}_{12j}-{\widehat{\overline{Y}}}_{12}\right)\left({X}_{12j}-{\widehat{\overline{Y}}}_{12}\right)}{\left({n}_{12}-1\right)} $$

$$ {s}_{X_{12}}^2=\frac{{\displaystyle {\sum}_j^{n_{12}}}{\left({X}_{12j}-{\widehat{\overline{X}}}_{12}\right)}^2}{\left({n}_{12}-1\right)} $$

*X*_*12j*_ = measurement of permanent plot *j* at time 1, *j* = 1,…, *n*_*12*_

*Y*_*12j*_ = measurement of permanent plot *j* at time 2, *j* = 1,…, *n*_*12*_

(2) For both means, the variance is calculated.

6 $$ V\left({\widehat{\overline{Y}}}_I\right)={s}_{Y.X}^2\left[\frac{1}{n_{12}}+\frac{{\left({\widehat{\overline{X}}}_1-{\widehat{\overline{X}}}_{12}\right)}^2}{{\displaystyle {\sum}_j^{n_{12}}}{\left({X}_{12j}-{\widehat{\overline{X}}}_{12}\right)}^2}\right]+\frac{s_{Y_{12}}^2-{s}_{Y.X}^2}{n_1} $$

where

$$ {s}_{Y_{12}}^2=\frac{{\displaystyle {\sum}_j^{n_{12}}}{\left({Y}_{12j}-{\widehat{\overline{Y}}}_{12}\right)}^2}{\left({n}_{12}-1\right)} $$

$$ {s}_{Y.X}^2=\frac{\left(1-{r}^2\right){\displaystyle {\sum}_j^{n_{12}}}{\left({Y}_{12j}-{\widehat{\overline{Y}}}_{12}\right)}^2}{\left({n}_{12}-2\right)}=\mathrm{mean}\ \mathrm{squared}\ \mathrm{error}\ \mathrm{of}\ \mathrm{the}\ \mathrm{regression} $$

$$ \mathrm{r}=\frac{s_{XY}}{S_{X_{12}}{S}_{Y_{12}}}=\mathrm{estimated}\ \mathrm{correlation}\ \mathrm{between}\ \mathrm{o}\mathrm{bservations}\ \mathrm{at}\ \mathrm{time}\ 1\ \mathrm{and}\ 2\ \mathrm{f}\mathrm{o}\mathrm{r}\ \mathrm{the}\ {n}_{12}\ \mathrm{plots} $$

7 $$ V\left({\widehat{\overline{Y}}}_{II}\right)=\frac{s_{Y_{-2}}^2}{n_{-2}}=\frac{{\displaystyle {\sum}_J^{n_{-2}}}{\left({Y}_{-2j}-{\widehat{\overline{Y}}}_{-2}\right)}^2}{n_{-2}\left({n}_{-2}-1\right)} $$

(3) Through weighting both means with their inverse variance, a combined estimator is derived. If the regression estimator has a larger variance, it therefore receives a lower weight and vice versa. These weights minimize the variance of the combined estimator.

8 $$ \widehat{\overline{Y}}=\frac{{\widehat{w}}_I{\widehat{\overline{Y}}}_I+{\widehat{w}}_{II}{\widehat{\overline{Y}}}_{II}}{\widehat{w}} $$

where

$$ {\widehat{w}}_i=\frac{1}{v\left({\widehat{\overline{Y}}}_i\right)} $$

$$ \widehat{w}={\widehat{w}}_I+{\widehat{w}}_{II} $$

(4) As the last step the variance of the combined estimator is calculated.

9 $$ V\left(\widehat{\overline{Y}}\right)=\left[1+\frac{4{\widehat{w}}_I{\widehat{w}}_{II}\left(\frac{1}{n_{12}-1}+\frac{1}{n_{-2}-1}\right)}{{\widehat{w}}^2}\right]/\widehat{w} $$

Once the estimates of the current mean $\widehat{\overline{Y}}$ and its variance V($\widehat{\overline{Y}}$) are computed, an estimation of change $\widehat{\overline{C}}$ and its variance V($\widehat{\overline{C}}$) can easily be obtained. The most straightforward estimation of change between 2 occasions is the combined current estimator, $\widehat{\overline{Y}}$, minus the mean calculated at previous occasion, ${\widehat{\overline{X}}}_I$

10 $$ \widehat{\overline{C}}=\widehat{\overline{Y}}-{\widehat{\overline{X}}}_I $$

An approximation of the variance of $\widehat{\overline{C}}$ is

11 $$ {V}_{SPR}\left(\widehat{\overline{C}}\right)=\frac{1}{\widehat{w}}+\frac{s_{X_1}^2}{n_1}-\frac{2\frac{{\widehat{w}}_I}{\widehat{w}}{\widehat{\beta}}_{YX}\ {\displaystyle {\sum}_j^{n_1}}{\left({X}_{1j}-{\widehat{\overline{X}}}_1\right)}^2}{\left({n}_1-1\right)} $$

While SPR is straightforward for 2 applications, estimation procedures become cumbersome for 3 or more occasions. For example, where SPR is applied for 3 occasions, 7 different plot types (*n*_*123*_ *, n*_*12−*_ *, n*_*1–3*_ *, n*_*−23*_ *, n*_*--3*_ *, n*_*1--*_ *, n*_*-2*-_) need to be considered [15].

### Methods and data

The comparison of the different sampling approaches renders the availability of information on population variances necessary. A long-term forest growth and yield experiment from Suriname was selected to provide the necessary information. The experiment, for which trees were initially measured in 1978 and remeasured in 2000 and 2013, was on a former concession site on which no forest management practices were applied since the establishment of the experiment. The basic treatments applied at that time were silvicultural treatments implemented at different intensity levels, and included release cuts – harvests similar to thinnings in temperate and boreal forests – to stimulate growth of the remaining stand. The treatments were allocated in 3 blocks, each containing 9 experimental plots. Each of the 9 experimental plots within a block was 1 hectare in size and surrounded by a buffer strip. In addition to the blocks, 3 plots were established in undisturbed natural forests, resulting in a total of thirty 1-ha plots.

Among the attributes assessed on the experimental plots was the tree diameter at breast height (dbh). [22] provided allometric functions that utilize regression models to convert dbh into an estimate of aboveground biomass (AGB). Among the AGB equations presented by Chave et al. [22], an equation was selected that achieved the smallest mean square error of prediction in a forested site in French Guyana, a neighboring country to Suriname. The equation has the form

12 $$ ln(AGB) = -1.562 + 2.148* ln(dbh) $$

and was utilized to estimate individual tree *AGB* based on measured *dbh* for the assessments in 2000 and 2013. A summary of tree level *AGB* values is found in Table 1. The large standard deviations are due to the typical inverse J-shaped distribution of trees by diameter class.

**Table 1:** **Tree level statsitics**

**Time**	**Number of trees**	**Above-ground biomass (** ***AGB*** **)**				
		**Min**	**Max**	**Mean**	**Median**	**Standard deviation**
		**[kg]**	**[kg]**	**[kg]**	**[kg]**	
2000	8650	70.1	9777.8	417.4	185.7	664.1
2013	8191	70.1	9581.5	485.3	223.9	717.1
		*DBH*				
		Min	Max	Mean	Median	Standard Deviation
		[cm]	[cm]	[cm]	[cm]	
2000	8650	15.0	149,1	29.3	23.6	16.4
2013	8191	15.0	147.7	31.6	25.7	17.4

The 1 ha experimental plots were further divided into 25 subplots with an area of 400 m^2^ each, resulting in 750 subplots in total. These subplots were used to calculate the input variances for studying the sampling design alternatives (Table 2). For this study, we do not consider the spatial allocation of the plots. The specification of sample sizes is a crucial element of designing a sample survey [19]. In order to facilitate the general understanding of the selected sampling design alternatives we applied the sample sizes presented in Table 3. At each occasion 375 plots are assessed. SPR was realized as a combination of 250 permanent (*n*_*12*_) plots and 125 temporary (*n*_*−1*_ *, n*_*−2*_) plots for occasions 1 and 2.

**Table 2:** **Plot statistics**

**Time**	**Number of plots**	**Above-ground biomass (** ***AGB*** **)**					
		**Min**	**Max**	**Mean**	**Median**	**Standard Deviation**	**Correlation**
		**[t/ha]**	**[t/ha]**	**[t/ha]**	**[t/ha]**		
2000	750	10.8	401.6	120.3	108.5	60.9	0.804
2013	750	17.5	542.7	132.5	118.6	65.5

**Table 3:** **Sample sizes**

**Plot type**	**Sampling design alternative**		
	**SPR**	**CFI**	**Temporary plots**
Temporary, time 1 (*n*_*1−*_)	125		375
Permanent (*n*_*12*_)	250	375	
Temporary, time 2 (*n*_*−2*_)	125		375

6 scenarios were applied to the plots and analyses were conducted with the goal of showing the general behavior of the sampling design alternatives under different levels of forest degradation and deforestation. The scenarios were designed to reflect the effects of realistic deforestation and degradation activities. Due to the aspatial nature of the application of the treatments, they are a generalization of degradation and deforestation patterns, however they facilitate the understanding of the performance of the sampling design alternatives. The scenarios are presented in Table 4.

**Table 4:** **Deforestation and degradation scenarios**

**Scenario**	**Description**	**Anticipated degradation/deforestation pattern**
No intervention	Original plot data from both treated and untreated stands are used without modification	No degradation and deforestation activities
10% degradation, *dbh* < 35 cm	On 10 percent of the plots (*n* = 75) the biomass of trees with *dbh* < 35 cm was set to zero at occasion 2	Degradation by harvesting trees with small *dbh* for fuelwood
10% degradation, *dbh* > 45 cm	On 10 percent of the plots (*n* = 75) the biomass of trees with *dbh* > 45 cm was set to zero at occasion 2	Degradation by selectively harvesting trees with large dbh for timber procurement
20% degradation, *dbh* < 35 cm	On 20 percent of the plots (*n* = 150) the biomass of trees with *dbh* < 35 cm was set to zero at occasion 2	Degradation by harvesting trees with small dbh for fuelwood
20% degradation, *dbh* > 45 cm	On 20 percent of the plots (*n* = 150) the biomass of trees with *dbh* > 45 cm was set to zero at occasion 2	Degradation by harvesting trees with large dbh for timber procurement
5% deforestation	On 5 percent of the plots (*n* = 37) the biomass of all trees is set to zero at the second occasion	Deforestation and land-use change

Numerical results of the combinations of scenarios and sample design alternatives were obtained by a Monte Carlo experiment. The experiment was realized with 1000 iterations for each combination. The 750 sample plots served as input for the simulations. In each iteration plots were randomly selected (simple random sampling without replacement) for applying the treatments of the 6 scenarios. In addition the original measurement values were maintained in order to provide input for the realizations with treatment bias. From the modified set of plots (n = 750) samples were selected by simple random sampling without replacement according to the sample sizes and plot types (permanent, temporary) given in 0. For each iteration population (true) values as well as sample estimates (current values and change between time 1 and time 2, and corresponding variances) were calculated.

The realizations of percentages of disturbance (deforestation, degradation) given in column 1 of Table 4 refer to the entire population of 750 plots, not to the selected samples. The original, undisturbed measurements at time 2 were utilized to simulate treatment bias. Hence, no degradation and deforestation activities take place on any of the plots assigned to the alternatives with treatment bias. Thereby, the endpoints of the conceivable range of treatment bias effects on permanent plots are depicted. Under realistic conditions, treatment bias will occur between these endpoints.

The Monte Carlo experiment was conducted in SAS**™**.
